# Clomiphene-Induced Severe Hypertriglyceridemia and Acute Pancreatitis: A Case Report and Literature Review

**DOI:** 10.7759/cureus.90836

**Published:** 2025-08-23

**Authors:** Zi Tan, Mortada Mohammed, Moneeb Mustafa, Lina Abdeldaim, Ammar Ahmed

**Affiliations:** 1 Endocrinology, Medical University of South Carolina Health Endocrinology at Nexton Medical Park, Summerville, USA; 2 Internal Medicine, Sentara Albemarle Medical Center, Elizabeth City, USA; 3 Internal Medicine, CHRISTUS St. Frances Cabrini Hospital, Alexandria, USA; 4 Internal Medicine, Rapid Regional Medical Center, Alexandria, USA; 5 Internal Medicine, Edward Via College of Osteopathic Medicine, Monroe, USA; 6 Internal Medicine, Mayo Clinic, Rochester, USA; 7 Medicine, University of Minnesota School of Medicine, Minneapolis, USA

**Keywords:** acute pancreatitis, clomiphene citrate, drug-induced dyslipidemia, fertility treatment, hypertriglyceridemia, ovulation induction, serm

## Abstract

Clomiphene citrate, a selective estrogen receptor modulator widely used for ovulation induction, has been rarely associated with severe hypertriglyceridemia, representing an under-recognized but potentially serious adverse effect. We present a case of clomiphene-induced severe hypertriglyceridemia complicated by acute pancreatitis in a 34-year-old woman with type 2 diabetes and baseline moderate hypertriglyceridemia who presented with acute epigastric pain after two months of unprescribed use of clomiphene for fertility purposes. Laboratory investigations revealed severe hypertriglyceridemia of 6,576 mg/dL (normal <150 mg/dL), representing a 19-fold increase from her baseline level of 345 mg/dL five months prior, along with hyperglycemia (268 mg/dL), elevated HbA1c (10.2%), and imaging consistent with acute pancreatitis secondary to hypertriglyceridemia. The patient was treated with aggressive fluid resuscitation and continuous insulin infusion, resulting in a reduction in triglycerides to 255 mg/dL within three days, and clomiphene was permanently discontinued. She was discharged on a regimen of fenofibrate 145 mg daily and a total of 4 g of omega-3 fatty acids daily, and her triglyceride levels normalized to 288 mg/dL at discharge. This case highlights the importance of baseline lipid screening and monitoring in patients receiving clomiphene therapy, particularly those with pre-existing metabolic risk factors, and clinicians should maintain high clinical suspicion for drug-induced hypertriglyceridemia in patients presenting with acute pancreatitis while on clomiphene therapy, as immediate discontinuation and initiation of appropriate lipid-lowering treatment can lead to rapid improvement in triglyceride levels.

## Introduction

Clomiphene citrate is a selective estrogen receptor modulator (SERM) that has been a cornerstone of ovulation induction therapy since its FDA approval in 1967 [1]. As a first-line treatment for anovulatory infertility, clomiphene is prescribed to millions of women worldwide annually [2,3]. The drug functions by blocking estrogen receptors in the hypothalamus, thereby preventing negative feedback and increasing gonadotropin-releasing hormone (GnRH) secretion. This, in turn, stimulates the release of follicle-stimulating hormone (FSH) and luteinizing hormone (LH) [4].

While clomiphene is generally well-tolerated, recognized adverse effects include ovarian hyperstimulation syndrome, multiple pregnancies, visual disturbances, and mood changes [5,6]. However, metabolic complications, particularly severe dyslipidemia, remain underappreciated in clinical practice. The relationship between clomiphene and lipid metabolism is complex, as the drug exhibits both estrogenic and anti-estrogenic properties depending on the target tissue and baseline estrogen levels [7].

Estrogen's effects on lipid metabolism are well-established, typically resulting in decreased total cholesterol and low-density lipoprotein (LDL) levels while increasing high-density lipoprotein (HDL) and triglyceride concentrations [8,9]. Given clomiphene's SERM properties, similar metabolic effects are anticipated, although clinical evidence remains limited. To date, only three cases of clomiphene-induced severe hypertriglyceridemia have been reported in the literature [10-12], representing a significant knowledge gap in understanding this potentially profound adverse effect.

Recent advances in understanding SERM pharmacology and lipid metabolism mechanisms have provided new insights into how these drugs may precipitate dyslipidemia, particularly in patients with underlying metabolic risk factors [13,14]. The increasing use of fertility treatments and the growing prevalence of metabolic syndrome make recognition of this complication increasingly crucial for clinical practice.

We present the fourth reported case of clomiphene-induced severe hypertriglyceridemia, complicated by acute pancreatitis, and provide an updated review of the relevant literature with emphasis on risk factors, mechanisms, and management strategies.

## Case presentation

A 34-year-old woman presented to the emergency department of Unity Hospital, Rochester, New York, USA, with a four-day history of severe epigastric pain radiating to her back, accompanied by nausea and multiple episodes of vomiting. The pain was described as sharp, constant, and rated eight out of 10 in severity.

The patient had a medical history significant for type 2 diabetes mellitus diagnosed five years prior, class I obesity (BMI 32 kg/m²), and moderate hypertriglyceridemia. Her diabetes had been poorly controlled despite multiple adjustments to her medication. Family history was notable for diabetes mellitus in her maternal aunt and brother, with no known family history of hyperlipidemia, premature atherosclerosis, hepatic disease, or pancreatic disorders.

Her current medications included atorvastatin 20 mg daily, metformin 1000 mg twice daily, insulin glargine 24 units daily, and omeprazole 20 mg daily. Of particular importance, the patient revealed she had been self-administering clomiphene citrate obtained online without a prescription for approximately two months prior to presentation. She was uncertain of the exact dosage but estimated taking "one tablet daily" in an attempt to conceive.

Her vital signs were stable (blood pressure 128/82 mmHg, heart rate 88 bpm, temperature 98.6°F, oxygen saturation 98% on room air). Abdominal examination revealed a soft abdomen with decreased bowel sounds and generalized tenderness most pronounced in the epigastric and right upper quadrant regions, without rebound tenderness or guarding. No hepatosplenomegaly was appreciated. The remainder of the physical examination was unremarkable.

Initial laboratory studies revealed several significant abnormalities as detailed in Table 1.

**Table 1:** Laboratory results throughout the patient's clinical course Values in bold indicate severe abnormalities; LDL-C: Low-density lipoprotein cholesterol; HDL-C: High-density lipoprotein cholesterol; WBC: White blood cell

Parameter	Normal range	One year earlier	Five months earlier	Presentation	Discharge (day 3)
Serum chemistry					
Sodium (mEq/L)	136-145	138	137	133	139
Potassium (mEq/L)	3.5-5.1	5.5	4.3	2.9	3.3
Anion gap (mEq/L)	7-16	17	15	10	7
Creatinine (mg/dL)	0.5-1.1	0.61	0.62	0.6	0.88
Glucose (mg/dL)	60-140	322	308	268	182
Albumin (g/dL)	3.5-5.2	4.4	4.6	4	-
Pancreatic enzymes					
Lipase (U/L)	6-51	-	-	30	-
Amylase (U/L)	30-110	-	-	85	-
Inflammatory markers					
WBC (×10³/μL)	4.0-11.0	-	-	17	8.2
Lipid panel & diabetes					
HbA1c (%)	4.2-5.6	11.4	-	10.2	-
Total cholesterol (mg/dL)	<200	184	220	636	245
Triglycerides (mg/dL)	<150	288	345	6,576	288
HDL-C (mg/dL)	40-60	38	38	15	42
LDL-C (mg/dL)	<100	88	113	Not calculable	95
Ketones					
β-hydroxybutyrate (mmol/L)	0.02-0.27	-	-	0.84	0.15

Most notably, the triglyceride level was markedly elevated at 6,576 mg/dL (normal <150 mg/dL), representing a dramatic increase from the 345 mg/dL documented five months earlier. Additional findings included leukocytosis (17.0 × 10³/μL), hypokalemia (2.9 mEq/L), hyperglycemia (268 mg/dL), mild ketosis (β-hydroxybutyrate 0.84 mmol/L), and severe hypercholesterolemia (636 mg/dL). Notably, HbA1c was elevated at 10.2%, indicating poor long-term glycemic control. Liver enzymes, lipase, and pancreatic amylase levels remained within normal limits.

**Figure 1:**
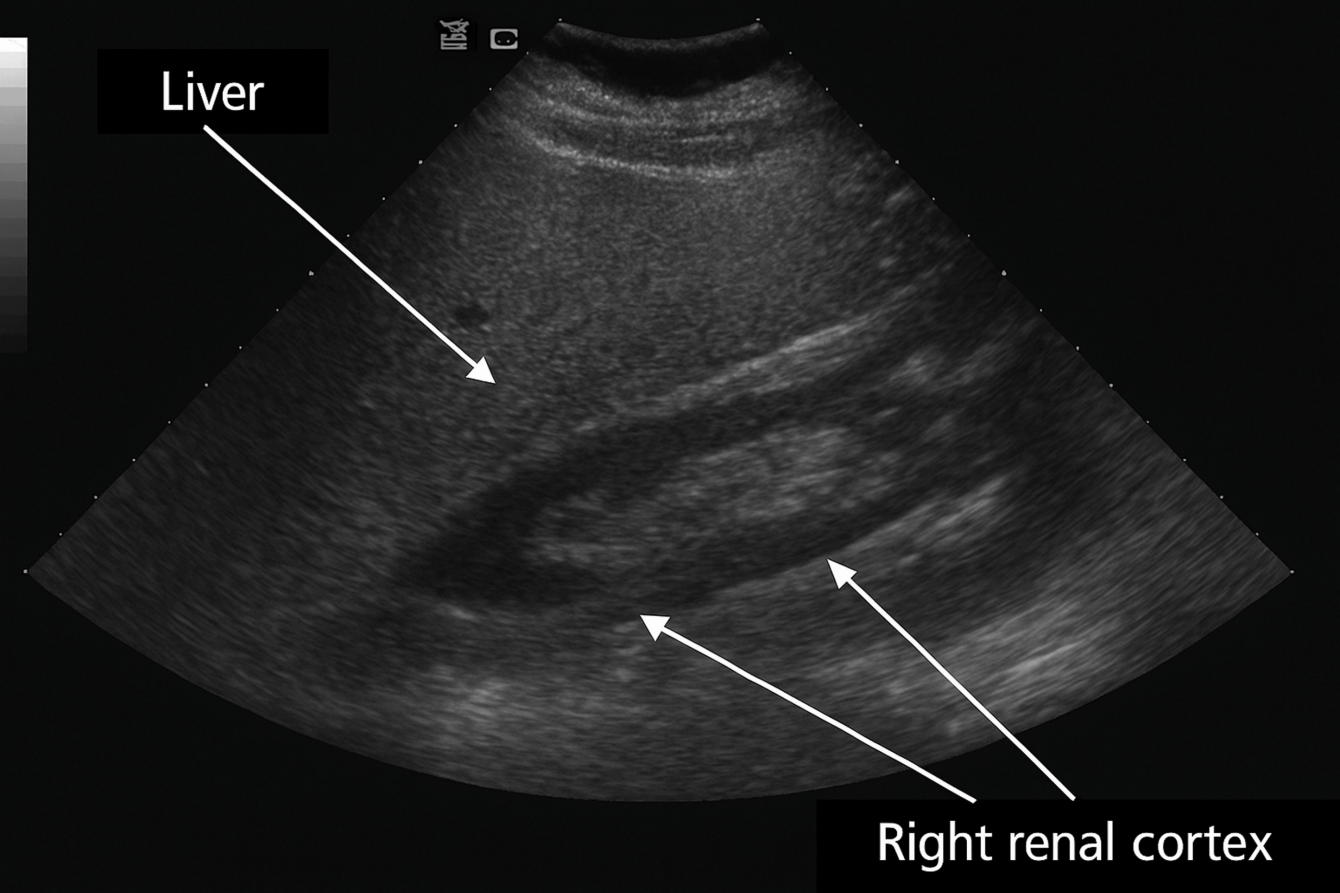
Abdominal ultrasound demonstrating hepatic steatosis Grayscale abdominal ultrasound image showing increased echogenicity of the liver parenchyma (labeled) compared to the adjacent right renal cortex, consistent with hepatic steatosis. The increased echogenicity obscures vascular markings, which is typical of fatty infiltration.

Abdominal ultrasonography demonstrated fatty infiltration of the liver (Figure 1).

**Figure 2:**
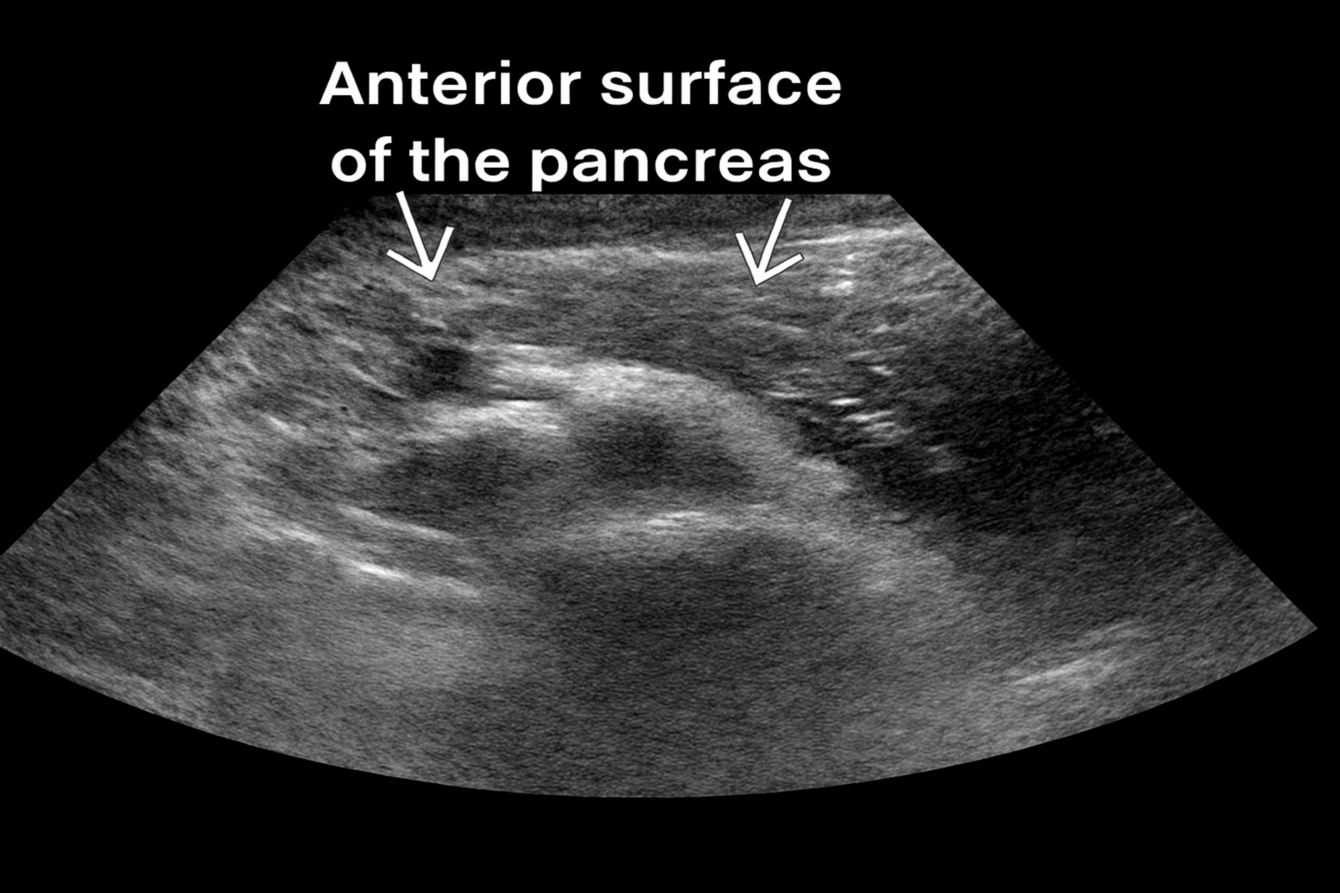
Abdominal ultrasound demonstrating pancreatic echotexture Grayscale ultrasound image displaying a heterogeneous echotexture of the anterior surface of the pancreas with increased echogenicity, suggestive of acute pancreatitis. No evidence of ductal dilatation or peripancreatic fluid collections was observed.

The pancreas appeared heterogeneous with increased echogenicity, consistent with acute inflammatory changes (Figure 2).

No pancreatic ductal dilatation or fluid collections were identified.

Based on the constellation of severe hypertriglyceridemia, characteristic abdominal pain, and imaging findings, the patient was diagnosed with hypertriglyceridemia-induced acute pancreatitis. Treatment was initiated with aggressive intravenous fluid resuscitation (normal saline at 200 mL/hour) and continuous insulin infusion (10 units/hour) to reduce triglyceride levels rapidly.

The patient showed an excellent response to therapy, with triglyceride levels decreasing to 255 mg/dL within 72 hours of treatment initiation, as shown in Table 1. Her abdominal pain gradually resolved, and she was able to tolerate oral intake without difficulty. Given the temporal relationship between clomiphene initiation and the development of severe hypertriglyceridemia, the drug was permanently discontinued.

The patient was discharged on day three of hospitalization with triglyceride levels of 288 mg/dL. Discharge medications included fenofibrate 145 mg daily and omega-3 fatty acids 4 g daily for ongoing lipid management, in addition to her previous medications for diabetes. She was strongly advised against future clomiphene use and counseled regarding proper medical evaluation for fertility concerns. Unfortunately, the patient was lost to follow-up despite scheduled endocrinology appointments.

## Discussion

This case represents the fourth reported instance of clomiphene-induced severe hypertriglyceridemia in the medical literature, highlighting an underappreciated but potentially serious adverse effect of this commonly prescribed fertility medication. The dramatic 19-fold increase in triglyceride levels from baseline, coupled with the temporal relationship to clomiphene initiation, strongly suggests a causal relationship between drug exposure and the development of severe dyslipidemia.

Clomiphene citrate exerts its therapeutic effects through complex interactions with estrogen receptors throughout the body [15]. As a SERM, clomiphene demonstrates tissue-selective estrogen receptor modulation, acting as an estrogen antagonist in the hypothalamus while exhibiting agonist properties in other tissues [16]. This dual mechanism explains both its therapeutic efficacy in ovulation induction and its potential for diverse metabolic effects. The relationship between SERMs and lipid metabolism has been extensively studied with newer agents such as tamoxifen and raloxifene [17,18]. These medications consistently demonstrate effects on triglyceride levels, particularly in patients with an underlying genetic predisposition to hypertriglyceridemia [19]. The proposed mechanism involves estrogen-like effects on hepatic lipid metabolism, including increased hepatic very low-density lipoprotein (VLDL) synthesis and decreased lipoprotein lipase activity, resulting in reduced triglyceride clearance [20].

Recent molecular studies have identified specific pathways through which SERMs may influence lipid metabolism. The peroxisome proliferator-activated receptor (PPAR) pathway, critical for lipid homeostasis, appears to be modulated by SERMs in a tissue-specific manner [16]. Additionally, apolipoprotein gene expression, particularly that of ApoC-III, may be upregulated by SERMs, contributing to hypertriglyceridemia through the inhibition of lipoprotein lipase [17]. An analysis of the four reported cases, including the present patient, reveals several common risk factors for clomiphene-induced hypertriglyceridemia. All patients had baseline metabolic abnormalities, including diabetes mellitus, pre-existing dyslipidemia, or obesity [10-12]. This suggests that patients with underlying metabolic dysfunction may be at increased risk for severe lipid derangements when exposed to clomiphene.

The role of genetic predisposition cannot be overstated. Recent advances in pharmacogenomics have identified genetic variants that affect lipid metabolism, potentially predisposing individuals to drug-induced hypertriglyceridemia [10]. Mutations in genes encoding lipoprotein lipase, apolipoprotein C-II (APOC2), apolipoprotein A-V (APOA5), and other enzymes involved in lipid metabolism have been associated with severe hypertriglyceridemia, particularly when triggered by medications or metabolic stress [18]. The development of severe hypertriglyceridemia during clomiphene therapy has significant clinical implications. Triglyceride levels exceeding 1,000 mg/dL are associated with increased risk of acute pancreatitis, a potentially life-threatening complication [16]. In this case, the triglyceride level of 6,576 mg/dL placed the patient at extremely high risk for pancreatic complications, as evidenced by the heterogeneous pancreatic appearance on ultrasonography (Figure 1).

Current management of severe hypertriglyceridemia focuses on rapid triglyceride reduction through multiple therapeutic modalities. Insulin therapy, as employed in this case, effectively reduces triglyceride levels by activating lipoprotein lipase and inhibiting hormone-sensitive lipase [13]. Apheresis may be considered for triglyceride levels exceeding 5,000 mg/dL; however, it was not required in this case, as a rapid response to medical therapy was observed [15]. Long-term management involves a combination of lipid-lowering therapy with fibrates and omega-3 fatty acids, both of which were initiated in this patient [16]. Fenofibrate, in particular, has demonstrated efficacy in reducing triglyceride levels and preventing recurrent pancreatitis in patients with severe hypertriglyceridemia [20].

The rarity of reported cases may reflect under-recognition rather than true incidence, suggesting the need for enhanced clinical awareness and monitoring protocols. Current fertility treatment guidelines do not routinely recommend lipid monitoring during clomiphene therapy [15]. However, based on the available evidence, several recommendations emerge. All patients should undergo a comprehensive metabolic evaluation before initiating clomiphene, including a fasting lipid profile, assessment of glucose homeostasis, and evaluation of family history for lipid disorders [14]. Patients with diabetes, obesity, pre-existing dyslipidemia, or a family history of lipid disorders should be considered high-risk and monitored more frequently [16]. Monthly lipid panels are recommended during the first three months of therapy, with subsequent monitoring every three months for ongoing treatment, as this appears prudent [18]. Clear communication regarding symptoms of hypertriglyceridemia and pancreatitis, with instructions for immediate medical evaluation if symptoms develop, is essential.

The landscape of fertility treatment has evolved significantly since clomiphene's introduction, with several newer options offering potentially improved safety profiles. Letrozole, an aromatase inhibitor, has emerged as an effective alternative for ovulation induction with different metabolic effects [15]. Gonadotropin therapy, while more complex to administer, may be preferable in patients with significant metabolic risk factors [17]. The absence of genetic testing in this case limits the ability to identify specific mutations associated with hypertriglyceridemia. Furthermore, the patient's loss to follow-up prevented evaluation of long-term outcomes and triglyceride levels following complete drug washout. Finally, the uncertain dosage of clomiphene, due to its unprescribed use, hinders interpretation of any potential dose-response relationship.

## Conclusions

This case highlights the potential for clomiphene citrate to cause severe, life-threatening hypertriglyceridemia, particularly in patients with underlying metabolic risk factors. Healthcare providers prescribing clomiphene should implement comprehensive metabolic screening and monitoring protocols. Early recognition, along with prompt management, can prevent serious complications.

As fertility treatments become increasingly accessible, enhanced awareness of metabolic complications is essential for optimizing patient safety. Future research should focus on identifying predictors of drug-induced hypertriglyceridemia to enable personalized risk assessment and prevention strategies.
